# Supplementary material for: A Qualitative Study on Concerns, Needs, and Expectations of Hospital Patients Related to Climate Change: Arguments for a Patient-Centered Adaptation
Source: Int J Environ Res Public Health. 2021 Jun 5;18(11):6105. doi: 10.3390/ijerph18116105 (PMC8201225; doi:10.3390/ijerph18116105)
Supplement: Supplementary file 1 [file ijerph-18-06105-s001.zip › ijerph-1215303-supplementary.pdf]

CHARITÉ

CharitéCentrum für Innere Medizin und Dermatologie

Charité | Campus Mitte | 10098 Berlin

Medizinische Klinik m.S. Infektiologie und Pneumologie
Arbeitsbereich Ambulante Pneumologie
Leiter: Prof. Dr. med. Ch. Witt

Tel **(030) 450 565 022**
Fax (030) 450 565 922
pneumologie@charite.de

<u>Interview guide</u>

Date:

Interview-Number:

Geriatrics: Internal medicine: Internal medicine (Paediatrics): Psychiatry: Surgery:

- Obtain written informed consent
- Expected duration: 5-15 minutes
- Resolve open questions

Hello. We are examining the expectations of patients in respect to the future hospital treatment against the background of climate change. Extreme weather such as heatwaves, stronger storms or droughts will occur more frequently in the future.

Please do not state your name, your exact age or where you are living during the interview

-What age group are you in? A) < 21 B) 21-30 C) 31-40 D) 41-50 E) 51-60 F) 61-70 G) 71-80 H) 81-90 I) > 90 ϒ

-Which gender are you? A) female B) male C) not specified

1) Relationship between health and climate change

1. Do you see a link between health and climate change or more extreme weather events? Yes No

2. To what extent will climate change, e.g. the occurrence of more frequent heatwaves, heavier storms or prolonged periods of drought affect your personal health?

2) Expectations towards hospital treatment in the context of climate change

1. When you think about the effects of climate change, what do you expect from your hospital treatment in the future?

2. Think about your current hospital stay. What should change compared to the current hospital treatment due to climate change?

3. Think about your current treatment by doctors and nurses. Do you have special expectations of doctors or nursing staff due to climate change?

4. Think about the examinations you are currently undergoing. Do you have special expectations regarding the process of examinations due to climate change?

5. Think about the hospital you are in and your patient´s room. Do you have special expectations of patient´s rooms due to climate change

6. Do you wish to let us know something else or did any further questions arise?

That was the last question. Thank you for your participation.
